# Granulocytes Acquire Antiapoptosis Activity and Promote Tumor Growth during Tumor Progress

**DOI:** 10.1055/s-0041-1726335

**Published:** 2021-03-16

**Authors:** Han Li, Wenyan Shen, Yanjie Xu, Zien Wang, Linghao Wang, Zequn Ding, Zhiyuan Xie, Yan Zhang

**Affiliations:** 1 Med-X Research Institute & School of Biomedical Engineering, Shanghai Jiaotong University, Shanghai, People's Republic of China; 2 Department of Clinical Laboratory, Renji Hospital, School of Medicine, Shanghai Jiaotong University, Shanghai, People's Republic of China

**Keywords:** granulocyte, apoptosis, cancer

## Abstract

Granulocytes play important roles in cancer, and their apoptotic status is often changed by the influence of tumor environment. However, the changes and the function on granulocyte apoptosis in cancer are unclear. In this study, we used tumor-bearing mouse model and tumor patients to analyzed the apoptosis of granulocytes in different tissues by flow analysis and TUNEL fluorescence staining, and found that the percentage of apoptosis cells in granulocytes was significantly decreased in late-stage tumor-bearing mouse and patients. The in vitro co-culture experiment showed that these antiapoptotic granulocytes could significantly inhibit T cell proliferation, and RNA-seq proved that there was obvious difference on the transcriptome between these cells and control cells, particularly immune-related genes. What is important, adoptive transfer of these antiapoptotic granulocytes promoted tumor progress in mouse model. Conclusively, we found that granulocytes in late-stage tumor could delay the process of apoptosis, inhibit T cell proliferation, and acquire pro-tumor activity, which provides a new therapeutic target for tumor immunity.

## Introduction

For a long time, the cognition of granulocyte function was limited to nonspecific immunity of sterilization and pro-inflammatory. Recently, more and more studies have focused on the role of tumor-associated granulocytes in cancer biology. 1 In certain types of cancers, such as head and neck cancer and renal cell carcinoma, granulocytes infiltrating into the tumor are an effective indicator of prognosis and survival rate for patients with metastasis. 2 The antitumor effect of granulocytes is involved in different stages of tumor progression; however, the studies on the role of granulocytes in tumors are somewhat contradictory. Studies have shown that they cannot only inhibit tumor occurrence and development but also show the opposite effect.

In homeostasis, mature granulocytes will undergo spontaneous apoptosis in a short time to maintain the total number of granulocytes in the body. The apoptosis of granulocytes is of great significance for maintaining the homeostasis of the organism and eliminating inflammation. 3 4 Under certain inflammatory conditions, the apoptosis of granulocytes is accelerated, the proportion increases, and the life course of the cells changes. 5 6 However, there are not many studies on the apoptosis of granulocytes in tumors, and the function of granulocytes with altered apoptosis is unclear.

In this study, we found that the percentage of apoptotic granulocytes was dramatically increased in terminal stage of multiple tumors by using murine model and samples from patients. And the in vitro and in vivo experiments showed the immunosuppressive activity and protumor activity of these antiapoptic granulocytes.

## Results

### The Percentage of Apoptotic Granulocytes Decreased during Tumor Progression

First, we used AKR esophagus cancer mouse model to examine the percentage of apoptosis cells in total myeloid cells during tumor progression. FCM analysis was used to investigate the percentage of CD11b ^+^ myeloid cell and its apoptotic rate in spleen, peripheral blood, and tumor infiltrating leucocytes (TILs) after 2 weeks (early stage) and 4 weeks (late stage) of tumor implantation. The results showed that the percentage of CD11b ^+^ myeloid cells in spleen, peripheral blood, and TILs were graduate increased along with the tumor progress ( Supplementary Fig. 1A and 2B [available in the online version]).

The annexin V and PI staining demonstrated that the percentage of apoptotic CD11b ^+^ myeloid cells was significantly reduced with tumor progress ( Supplementary Fig. 1C and 1D [available in the online version]). The phenotype that the number of CD11b ^+^ myeloid cells was increased, while the percentage of apoptotic myeloid cells was decreased along tumor growth was also observed in other types of tumors, such as 4T1-breast cancer ( Supplementary Fig. 2A and 2B [available in the online version]) and MC38-colorectal cancer ( Supplementary Fig. 2C and 2D [available in the online version]).

**Fig. 1:**
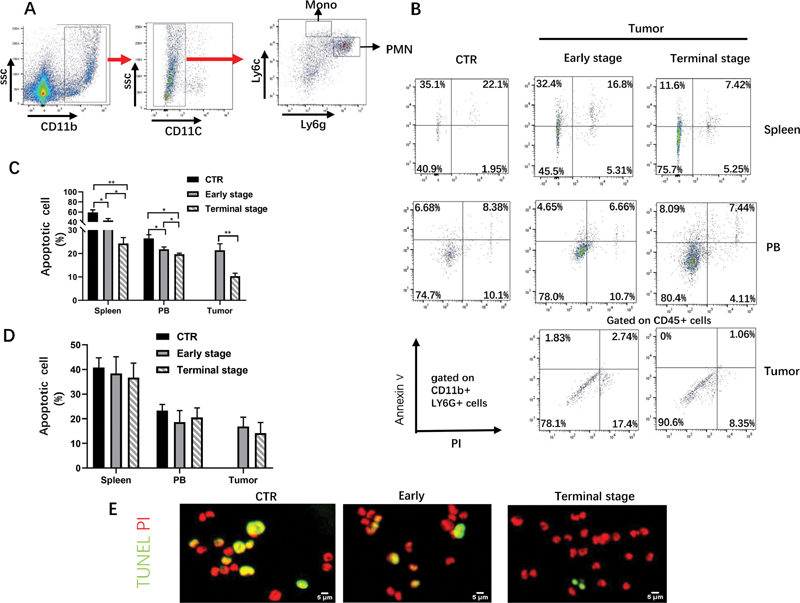
The majority of myeloid cells with reduced apoptosis rate are granulocyte. ( **A** ) Representative flow cytometry plot of the component of CD11b ^+^ myeloid cell. ( **B** ) Representative flow cytometry plot of apoptotic CD11b ^+^ Ly6g ^+^ cell percentage from AKR tumor-bearing and healthy C57/B6 mice in spleen, peripheral blood, and tumor tissue. ( **C** ) Quantitative statistical chart of B, *n* = 5 per group. ( **D** ) Quantitative statistical chart of apoptotic CD11b ^+^ Ly6c ^+^ cell percentage from AKR tumor-bearing and healthy C57/B6 mice in spleen, peripheral blood and tumor tissue. *n* = 5 per group. ( **E** ) TUNEL and PI staining of granulocyte in spleen from AKR tumor-bearing and healthy C57/B6 mice. * *p* < 0.05, ** *p* < 0.01, *** *p* < 0.001. Data shown as mean ± standard error of mean. AKR, aldo-keto reductase.

Since monocytes (MO) and granulocytes (PMN) are the two major subsets of myeloid cells, we further figured out if the percentage of apoptotic granulocytes was decreased along tumor growth. Granulocytes and monocytes were separated by Ly6g and Ly6c in CD11b ^+^ myeloid cells by FCM ( Fig. 1A ), and their apoptosis was examined by annexin V and PI. The results showed that the percentage of apoptosis in Ly6g ^+^ granulocytes was significantly decreased in spleen, peripheral blood, and TILs from tumor-bearing mice, compared with control group ( Fig. 1B and 1C ), while that of monocytes showed no significant differences among these three groups ( Fig. 1D ). To further verify the apoptotic status of granulocytes in tumor-bearing mice, TUNEL staining was performed on Ly6 g ^+^ granulocytes obtained by flow cytometry sorting. Similarly, the result of TUNEL demonstrated that the apoptosis rate of ly6g ^+^ granulocytes gradually decreased along with tumor growth ( Fig. 1E ). These results showed that the percentage of apoptotic granulocytes was significantly decrease during tumor progress.

### The Percentage of Apoptotic Granulocytes Decreased in Cancer Patients

**Fig. 2:**
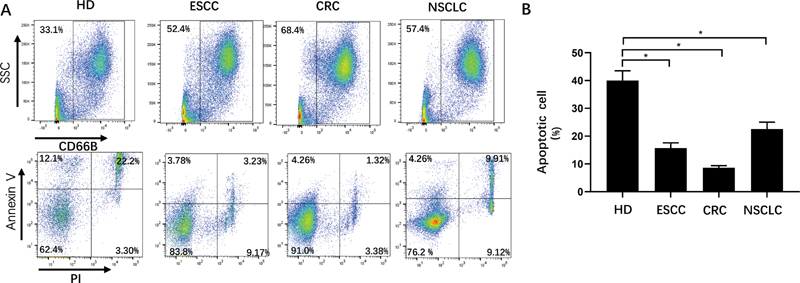
Apoptotic percentage of granulocytes from different kinds of cancer patients. ( **A** ) Representative flow cytometry plot of CD66B ^+^ granulocyte and its apoptotic percentage derived from the periphery blood of esophageal squamous cell carcinoma, colorectal cancer, nonsmall cell lung carcinoma patients and hemodialysis. ( **B** ) Analysis of Quantitative statistical chart of A. Each group contained five patients. * *p* < 0.05, ** *p* < 0.01, *** *p* < 0.001. Data shown as mean ± standard error of mean.

To further figure out whether there is also an inhibition on the proportion of apoptotic granulocytes in patients with advanced cancer, we collected immune cells from peripheral blood of patients with advanced esophageal cancer (ESCC), colorectal cancer (CRC) and nonsmall cell lung cancer (NSCLC). FCM was used to identify the percentage of CD11b ^+^ CD66b ^+^ granulocytes and its apoptosis rate, and the results showed that the percentage of CD11b ^+^ CD66b ^+^ granulocytes was significantly increased, and its apoptosis rate was decreased in these three types of cancer ( Fig. 2A and 2B ). These results showed that both tumor-bearing mice and cancer patients at terminal stage had more antiapoptotic granulocytes.

### Antiapoptotic Granulocytes (PMN ^TUMOR^ ) Inhibited the Proliferation of T Cells

**Fig. 3:**
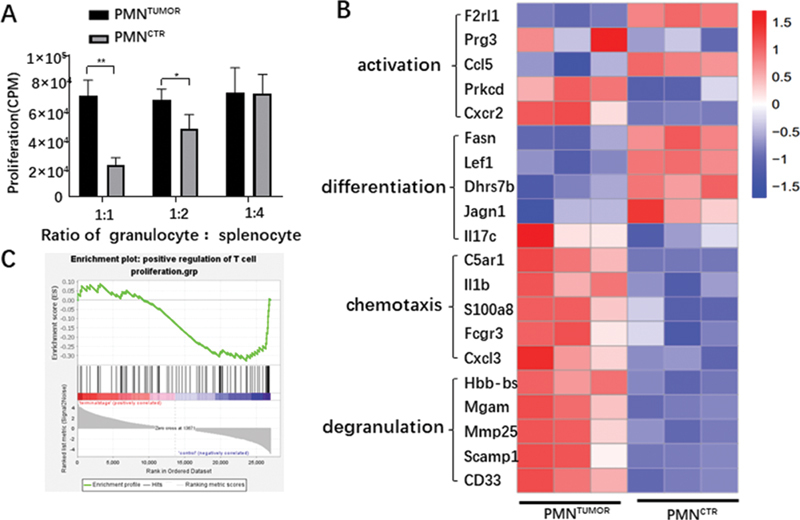
PMN ^TUMOR^ depress the proliferation of T cells. ( **A** ) Suppression of ovalbumin-activated T cell proliferation in PMN ^TUMOR^ isolated from AKR tumor-bearing C57/B6 in spleen, *n* = 3 per group. ( **B** ) Heatmap of granulocyte function related gene expression of PMN ^TUMOR^ and PMN ^CTR^ , *n* = 3 per group. ( **C** ) Gene set enrichment analysis enrichment analysis of positive regulation of T cell proliferation pathway. * *p* < 0.05, ** *p* < 0.01, *** *p* < 0.001. Data shown as mean ± standard error of mean. PMN, polymorphonuclear leukocyte.

Since previous studies showed that granulocytes in tumor, particularly at the terminal stage, could acquire the immunosuppressive activity, next we examined the effects of the antiapoptotic granulocytes (PMN ^TUMOR^ ) on the T cells proliferation. We respectively isolated PMN ^TUMOR^ and PMN ^CTR^ from splenocytes of tumor-bearing and control mice by FCM and co-cultured them with splenocytes acquired from OT-1 mice with different ratio. Fig. 3A showed that PMN ^TUMOR^ could significantly inhibit the proliferation of OVA-activated CD8 ^+^ T cells at ratio of 1:2 and 1:1, compared with PMN ^CTR^ . The transcriptional level of isolated PMN ^TUMOR^ and PMN ^CTR^ were also examined by RNAseq, and the results of heatmap showed that there was dramatically differential gene expression between PMN ^TUMOR^ and PMN ^CTR^ ( Fig. 3B ). Gene set enrichment analysis (GSEA) indicated that downregulated genes revealed low levels of expression in positive regulation of T cell proliferation in PMN ^TUMOR^ ( Fig. 3C ), which was consistent with immunosuppression effect in vitro. These results demonstrated that the antiapoptotic PMN ^TUMOR^ acquired the immunosuppressive activity.

### Antiapoptotic Granulocytes (PMN ^TUMOR^ ) Promoted Tumor Growth

**Fig. 4:**
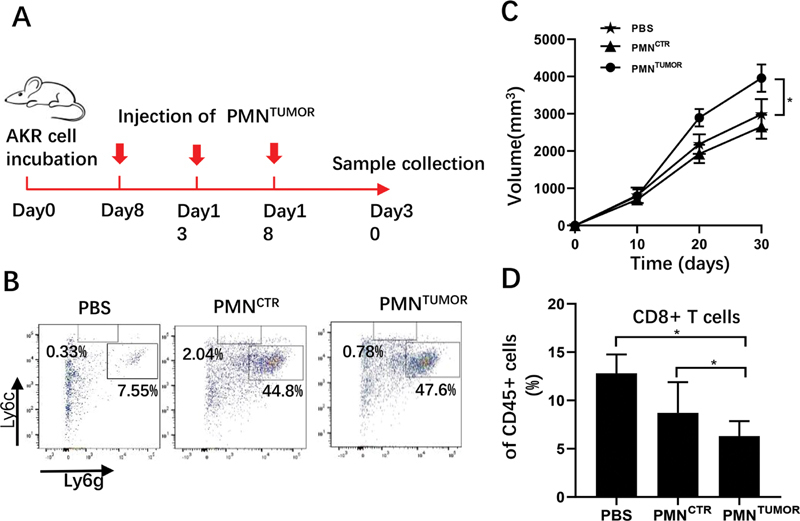
PMN ^TUMOR^ adoptive transfer promotes tumor growth. ( **A** ) Flow chart of experimental procedure. ( **B** ) Representative flow cytometry plot of CD11b ^+^ myeloid cell from AKR tumor-bearing mice injected with PMN ^TUMOR^ , PMN ^CTR^ , and PBS in peripheral blood. ( **C** ) The tumor growth curves of AKR tumor-bearing mice injected with PMN ^TUMOR^ , PMN ^CTR^ , and PBS. *n* = 5 per group. ( **D** ) Analysis of Quantitative statistical chart of percentage of CD8 ^+^ T cells in CD45 ^+^ cells obtained from mouse spleen injected with PBS, PMN ^CTR^ , and PMN ^TUMOR^ . * *p* < 0.05, ** *p* < 0.01, *** *p* < 0.001. Data shown as mean ± standard error of mean. AKR, aldo-keto reductase; PBS, phosphate-buffered saline; PMN, polymorphonuclear leukocyte.

To further confirm the protumor activity of PMN ^TUMOR^ in vivo, we adoptively transferred 1 × 10 ^6^ PMN ^TUMOR^ or PMN ^CTR^ to tumor-bearing mice on day 8, 13, and 18 after tumor implantation ( Fig. 4A ). Peripheral blood was drawn from mice caudal vein on day 15 to identify the efficiency of adoptive transfer, and the results of flow cytometry showed that, compared with control group (PBS injection), there were a lot of Ly6g ^+^ granulocytes accumulated in PMN ^TUMOR^ and PMN ^CTR^ groups ( Fig. 4B ), suggesting that both PMN ^TUMOR^ and PMN ^CTR^ were successfully transferred into tumor-bearing mice. During adoptive transfer experiment, tumor volume was recorded on day 10,20,30, and the tumor growth curves showed that the tumors of mice injected with PMN ^TUMOR^ grew faster than those of the other two groups ( Fig. 4C ). We also analyzed the percentage of CD8 ^+^ T cells in TILs among those three groups, and the results of FAM demonstrated that the percentage of CD8 ^+^ T cells in TILs was significantly decreased in PMN ^TUMOR^ group, compared with PBS and PMN ^CTR^ group ( Fig. 4D ). These results suggested that PMN ^TUMOR^ could promote tumor growth by inhibiting T cells proliferation.

## Discussion

When granulocytes enter an apoptotic state, they will gradually lose the ability to carry out physiological activities such as chemotaxis, respiratory burst, and degranulation. 7 For some subsets of granulocytes, such as eosinophils, the delay in apoptosis can lead to the activation of chronic inflammation and autoimmune related pathways. 8 In some inflammatory states, the apoptosis of granulocytes will be delayed. 4 Similarly, the life course of granulocytes changes in different cancer types, and the process of apoptosis is also affected differently. In melanoma, the proportion of apoptotic granulocytes decreased, showing a state of delayed apoptosis. 9 However, there was no significant changes in the proportion of apoptosis in granulocytes and the expression of apoptosis-related pathways in patients with kidney cancer. 10 Our research found that in the advanced stages of multiple tumor types, such as esophageal cancer, nonsmall cell lung cancer and colorectal cancer, the proportion of apoptotic granulocytes significantly decreased in both mouse model and clinical patients. In the early stage of tumor, the delayed apoptosis in granulocytes was not significant, which may explain why some tumor types do not observe obvious changes on apoptosis of granulocytes.

Previous studies showed that many of the pro-tumor activities of granulocytes were similar as those of granulocytes (G) subset of bone marrow derived suppressor cells (MDSC), and so far there is no specific cell surface markers to distinguish these two different subsets. Fridlender et al 11 has compared the transcriptomes of granulocytes and G-MDSC acquired from tumor-bearing mouse model and found that there was significantly difference between these two subsets on the transcriptome level of genes related to granule protein, respiration, cytoskeleton, and apoptosis. Our research demonstrated that the transcriptome of granulocytes and normal granulocytes in the terminal stage of tumor have changed significantly, especially the immune-related genes. Further studies have found that these granulocytes could significantly inhibit the proliferation of T cells, particularly in the adoptive transfer experiment, and significantly promote the tumor growth in mouse model.

Our studies have demonstrated that characteristics of reduced apoptosis of granulocytes in the terminal stage of tumors, and showed that granulocytes with delayed apoptosis acquired the pro-tumor activity. These results suggest that targeting the apoptosis pathway of granulocytes may be a promising new direction for tumor immunotherapy.

## Materials and Methods

### Clinical Specimens

Samples of patients and healthy donators were obtained from Chest Hospital and Renji Hospital affiliated to Shanghai Jiao Tong University. Human study protocols were in accordance with the Declaration of Helsinki. All volunteers signed the written informed consent forms.

### Cell Culture and Isolation

Mouse cancer cell lines were kept in our laboratory or purchased from the Qingqi Biotechnology Development Co. Ltd (Shanghai, China). All cells were cultured in DMEM (Gibco, United States) supplemented with 10% fetal bovine serum (Excel, China) at 37°C under 5% CO _2_ .

### Xenograft Tumor

All animal experiments were performed according to the regulations of Laboratory Animal Welfare and Ethics Committee of Shanghai Jiao Tong University. Female mice (6–8 weeks old) were obtained from Jiesijie Laboratory Animal Co., Ltd (Shanghai, China). 10 ^6^ tumor cells were injected subcutaneously into the thighs of 6 to 8 weeks of female mice.

### Single Cell Suspension

Tumors were dissected, minced, and digested with cell dissociation solution, containing 2 mg/mL collagenase IV (Thermo Fisher Scientific), 0.75 mg/mL hyaluronidase (Sigma–Aldrich, Missouri, United States), and 0.2 mg/mL DNase I (Roche, Mannheim, Germany) in PBS for 2 hours at 37°C shaking incubators. Enzymatic reactions were stopped by addition of enough 1 × PBS, and suspensions were dispersed through a 40-μm cell strainer. Cells were collected by centrifugation. 1 × lysing buffer (BD Biosciences) was added to remove red blood cells. The spleen was removed into a dish and smashed gently by cell scraper then the suspension was filtered through a 40 µm cell strainer. Peripheral blood was collected in anticoagulant tube and then was mixed with 1 × lysing buffer to remove red blood cells.

### Flow Cytometry Analysis and Sorting

In total, 100 μL cell suspension (10 ^7^ cells/mL) were collected by centrifugation, followed by staining with 5 μL percp-cy5.5 CD11b, Pacific Blue ly6 g, APC-cy7 ly6c, or APC CD11c antibody (BD Biosciences). The mixture was incubated for 15 to 30 minutes on ice in the dark. Cells were washed twice and collected by centrifugation. The cells were then resuspended in binding buffer and stained for 15 minutes in the dark with annexin V and PI (Annexin V-FITC Apoptosis Detection Kit; BD Biosciences). LSRFortessa flow cytometer (BD Biosciences) was used to analyze the cells. Data were analyzed by using FlowJo-V10 software (Tree Star, Ashland, Oregon, Unite States). Cells were stained as described above and sorted on BD FACS Aria (BD Biosciences).

### TUNEL Staining

TUNEL staining (TUNEL Assay Kit, Abcam, Cambridge, England) was used to observe the apoptosis extent of cells. Cell suspension (2 × 10 ^6^ cells/mL) was collected by centrifugation. Then the cells were fixed with 1% paraformaldehyde on ice for 15 minutes, collected by centrifugation and washed with 1 × PBS twice. Next, the cells were fixed with 70% ethanol on ice for 30 minutes, collected by centrifugation and washed with wash buffer twice. Cells were resuspend in DNA Labeling Solution for 60 minutes at 37°C. Rinse buffer was added to end the staining process. Cell suspension was collected by centrifugation, resuspended in propidium iodide/RNase A Solution, and incubated in the dark for 30 minutes at room temperature. Cells were dropped onto glass slide coated with poly-lysine. Finally, the cells were observed with a fluorescent microscope (Leica, Wetzlar, Germany).

### T Cell Co-Culture

Single-cell suspensions from spleen were stained and sorted on BD FACS Aria (BD Biosciences). CD11b ^+^ Ly6G ^+^ Ly6C ^−^ PMN ^TUMOR^ were plated in dishes in 1,640 RPMI with 10% FBS and co-cultured at different ratios with splenocytes from OT-itransgenic mice in the presence of 0.1 ng/mL ovalbumin (Sigma–Aldrich). After 48 hours, cells were incubated with (3H) thymidine (PerkinElmer, Waltham, United States) for 16 to 18 hours. Proliferation was quantized by using the TopCount NXT instrument (PerkinElmer).

### RNA-Seq

Total RNA was isolated with Trizol (Gibco) and then treated with DNase to digest genomic DNA. mRNA isolation was completed with NEB Next PolyA mRNA Magnetic Isolation Module (New England Biolabs, Ipswich, Massachusetts, United States). The RNA-Seq library was prepared with KAPA Stranded RNA-Seq Kit for Illumina (KAPA Biosystems, Wilmington, Massachusetts, United States). RNA-seq library was sequenced on Illumina HiSeq platform with paired-end 2 × 150 as the sequencing mode.

### Adoptive transfer

Resuspended PMN ^TUMOR^ and PMN ^CTR^ in ice-cold PBS at a concentration of 25 × 106 cells/mL and 200 μL of the suspension was injected into the lateral tails of tumor-bearing mice.
