# Supplementary material for: Granulocytes Acquire Antiapoptosis Activity and Promote Tumor Growth during Tumor Progress
Source: Glob Med Genet. 2021 Mar 16;8(2):72–7. doi: 10.1055/s-0041-1726335 (PMC8110362; doi:10.1055/s-0041-1726335)
Supplement: Supplementary file 1 — Supplementary Material [file 10-1055-s-0041-1726335-s2100010.pdf]

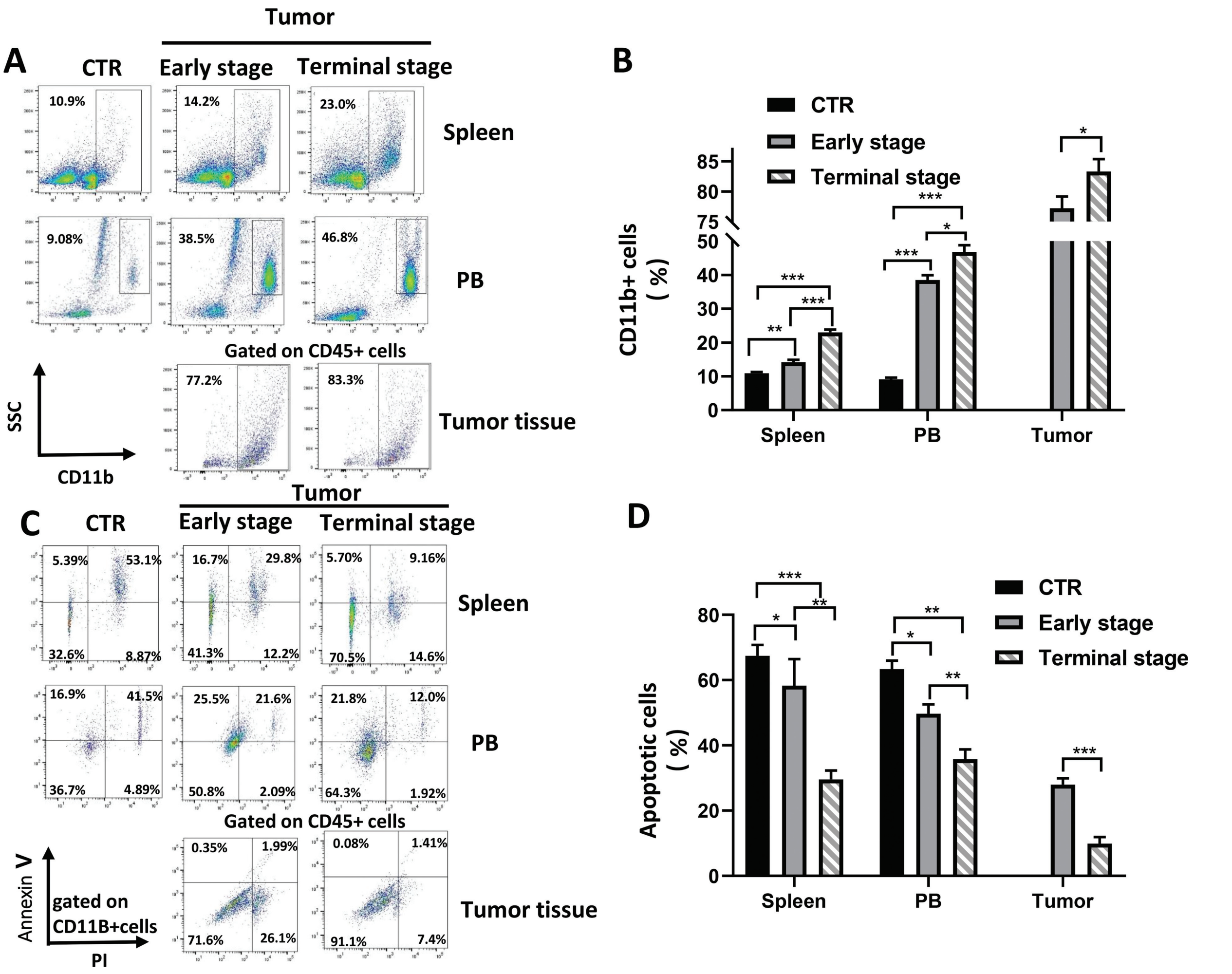

Supplementary Fig. S1 Percentage of apoptotic myeloid cells decrease with tumor growth. (**A**) Representative flow cytometry plot of $CD11b^{+}$ myeloid cells of aldo-keto reductase tumor-bearing and healthy C57/B6 mice in spleen and peripheral blood and tumor tissue. (**B**) Analysis of quantitative statistical chart of A. $n = 5$ per group. (**C**) Representative flow cytometry plot of apoptotic $CD11b^{+}$ cell percentage from AKR tumor-bearing and healthy C57/B6 mice in spleen, peripheral blood and tumor tissue. (**D**) Analysis of Quantitative statistical chart of C. $n = 5$ per group. $^{*}p < 0.05$, $^{**}p < 0.01$, $^{***}p < 0.001$; data shown as mean ± standard error of mean.

 © 2021. The Author(s).

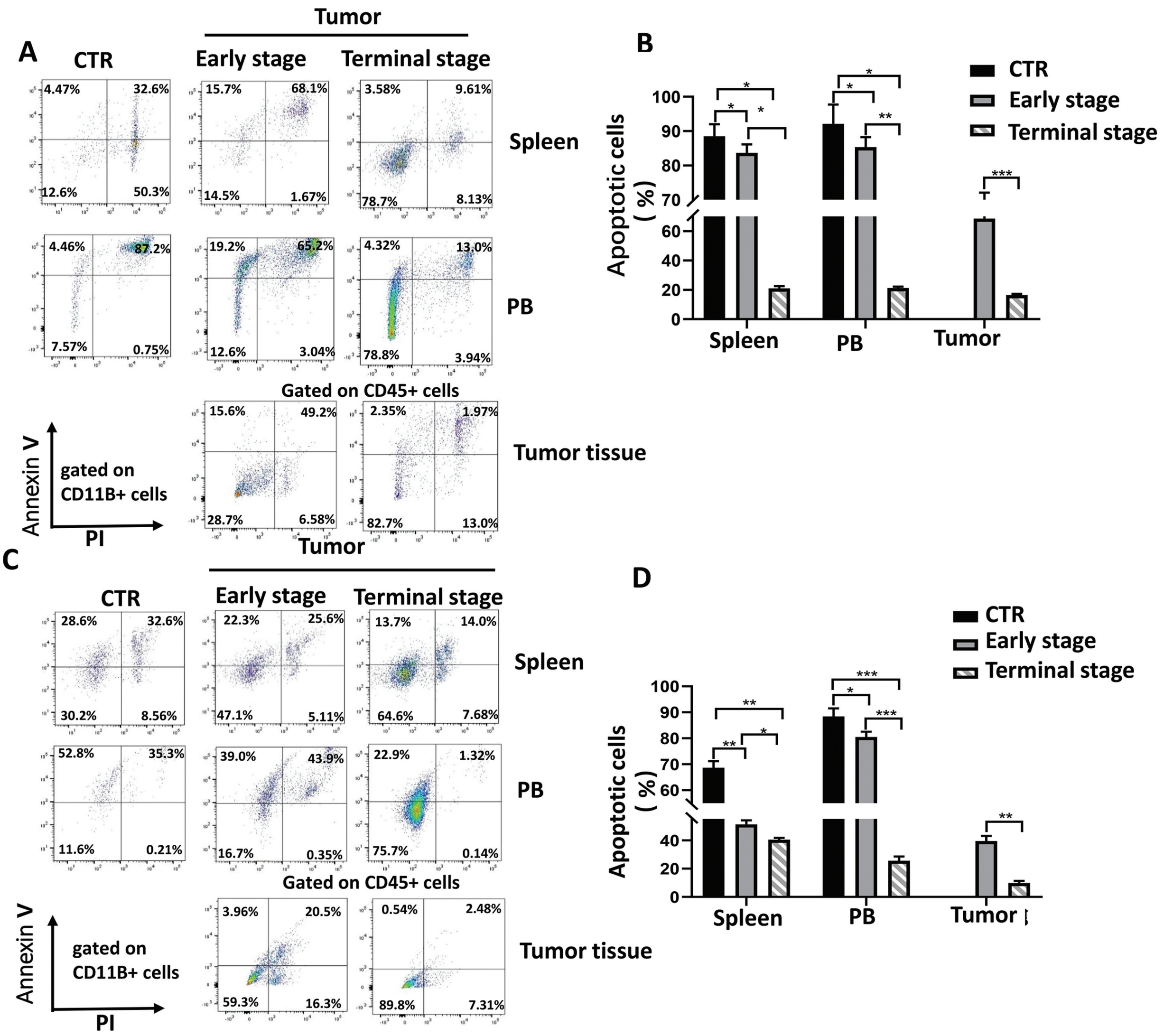

Supplementary Fig. S2 The phenomenon that apoptotic myeloid cells decrease with tumor growth arises in different cancer mouse model. (**A**) Representative flow cytometry plot of apoptotic CD11b^{+} cell percentage from 4T1 tumor-bearing and healthy Balb/C mice in spleen and peripheral blood and tumor tissue. (**B**) Analysis of Quantitative statistical chart of A. Each group contained five mice. (**C**) Representative flow cytometry plot of apoptotic CD11b^{+} cell percentage from MC38 tumor-bearing and healthy C57BL mice in spleen and peripheral blood and tumor tissue. (**D**) Analysis of quantitative statistical chart of C. Each group contained five mice. $^{*}p < 0.05$, $^{**}p < 0.01$, $^{***}p < 0.001$; data shown as mean ± standard error of mean.

 © 2021. The Author(s).
